# Proteome-wide characterization of *Plasmodium vivax* antigens using a high-density peptide array

**DOI:** 10.1371/journal.ppat.1014621

**Published:** 2026-09-28

**Authors:** Rosita Asawa, Brittany Hazzard, Katie Ko, Kieran Tebben, John Tan, Tineke Cantaert, Andrea Berry, Niraj Tolia, Jean Popovici, David Serre

**Affiliations:** 1 Institute for Genome Sciences, University of Maryland School of Medicine, Baltimore, Maryland, United States of America; 2 Nimble Therapeutics Inc., Madison, Wisconsin, USA; 3 Immunology Unit, Institut Pasteur du Cambodge, Phnom Penh, Cambodia; 4 Center for Vaccine Development, University of Maryland School of Medicine, Baltimore Maryland, United States of America; 5 Laboratory of Malaria Immunology and Vaccinology, National Institute of Allergy and Infectious Diseases, Bethesda Maryland, United States of America; 6 Malaria Research Unit, Institut Pasteur du Cambodge, Phnom Penh, Cambodia; 7 Infectious Disease Epidemiology and Analytics G5 Unit, Institut Pasteur, Paris, France; University of Cambridge, UNITED KINGDOM OF GREAT BRITAIN AND NORTHERN IRELAND

## Abstract

*Plasmodium vivax* is the second most prevalent *Plasmodium* species, with 2.5 billion people at risk of infection worldwide and around 10 million cases of clinical vivax malaria every year. Despite the clinical importance of this pathogen, little is known about the *P. vivax* proteins recognized by the host immune system, which hinders our ability to select vaccine candidates or to develop efficient serological markers. To characterize immunogenic *P. vivax* proteins, we designed a high-density peptide array containing 4.2 million peptides covering the entire protein sequences of all *P. vivax* genes and analyzed antibody responses of infected and malaria-naïve individuals. We identified a total of 283 proteins that are commonly immunogenic in symptomatic individuals. These proteins included many proteins known to be involved in erythrocyte invasion, several nucleoporins, and many uncharacterized proteins that should be further investigated for their roles during blood-stage infections. These analyses also revealed a unique pattern of antibody response against PIR proteins in asymptomatic individuals that could be associated with protection against clinical vivax malaria. Overall, these data provide an agnostic and proteome-wide perspective on immunogenic *P. vivax* proteins and constitute an important resource for the malaria community to develop new tools for better detecting and eliminating this important human pathogen.

## Author summary

*Plasmodium vivax* is the second most common cause of human malaria, threatening half of the world’s population. Despite the clinical importance of this pathogen, we only have a very limited number of vaccine candidates and diagnosis markers usable in the field, primarily due to our lack of global understanding of the parasite proteins recognized by the host immune system. To address this limitation, we designed a peptide array capturing the entire protein sequences of all *P. vivax* genes and used serum samples from infected individuals to identify which *P. vivax* proteins were bound by antibodies. Overall, we identified 283 commonly immunogenic proteins, including several exciting novel candidates for improving *P. vivax* detection and, possibly, developing efficient vaccines against vivax malaria.

## Introduction

Malaria is an infectious disease that affects millions of individuals each year [1]. It is caused by unicellular parasites of the *Plasmodium* genus, with *Plasmodium falciparum* and *Plasmodium vivax* responsible for more than 95% of the cases worldwide. *P. vivax* is the second most prevalent *Plasmodium* species, with 2.5 billion people at risk of infection and 10 million vivax malaria cases each year, primarily in Southeast Asia, India, and South America [1]. While *P. vivax* and *P. falciparum* both cause human malaria, these parasites have very different biological features [2,3]. For example, vivax malaria typically has a lower parasitemia compared to falciparum malaria, in part due to the preferential invasion of immature reticulocytes by *P. vivax* parasites [4,5] (while *P. falciparum* parasites can invade any red blood cell), a lower pyrogenic threshold [6], and less sequestration of mature stages in the circulation (but possibly more sequestration in the spleen [7,8]). In addition, *P. vivax* parasites have a hepatic stage not observed in *P. falciparum*: the hypnozoites. Hypnozoites can remain dormant in the liver for weeks to months after the initial infection has been cleared before reactivating to cause new blood stage infections [9]. This dormant stage allows *P. vivax* parasites to persist in areas where mosquitoes are present only seasonally, broadening the geographical distribution of the parasites, and facilitates parasite circulation, which complicates elimination and eradication efforts [10].

The limited sensitivity and specificity of the rapid diagnostic tests (RDTs) currently available for *P. vivax* and the lack of biomarkers for identifying latent hypnozoite burdens have limited our ability to efficiently identify and treat *P. vivax* infections [11,12]. Additionally, the lack of an *in vitro* culture system for *P. vivax* dramatically limits the scope of laboratory investigations that can be conducted on this important human pathogen. As a consequence, the therapeutic toolkit available to treat and prevent *P. vivax* infections remains limited and is primarily derived from drugs developed against *P. falciparum*. Artemisinin-based combination therapy and, in some regions, chloroquine are the current standard-of-care for the treatment of vivax malaria [1], but these treatments only target blood-stage parasites, leaving hypnozoites unaffected and able to cause relapses after successful treatment of the blood-stage infection [11,13]. Only primaquine and tafenoquine effectively target hypnozoites but the use of these drugs in endemic areas is complicated by severe side effects in G6PD-deficient individuals, a common trait geographically correlated with historical exposure to malaria [14]. Finally, while several promising vaccines against *P. falciparum* are in development [15], vaccine research on *P. vivax* has lagged behind, partially due to our limited understanding of *P. vivax* antigens: most studies thus far have focused on a handful of selected candidates primarily derived from the *P. falciparum* literature, with the exception of the Duffy binding protein (DBP) [16,17]. Unfortunately, the *P. vivax* vaccine candidates that are currently in early-stage clinical trials seem to only confer partial protection [18–23], highlighting the need to identify novel, evidence-based, candidates.

During a malaria infection, the host immune system mounts a response against the parasites, which involves the expansion of antigen-specific B and T cells, as well as the production of antibodies against specific parasite proteins [24,25]. Upon repeated infections, this response leads to the acquisition of immunity against the disease, if not sterilizing [26]. Understanding which parasite proteins are recognized by the immune system and elicit the production of a robust antibody response is key, not only to identifying new targets for vaccines, but also to developing biomarkers against specific stages (*e.g.*, the dormant hypnozoites) or of recent *Plasmodium* exposure [27–29]. However, the identification of antigenic molecules is complicated by the fact that *Plasmodium* parasites express thousands of different proteins at different times during their complex life cycle [30,31]. In addition, high-throughput synthesis of recombinant proteins for testing in serological assays is complicated by the complex structure of eukaryotic genes, which often contain introns (and can sometimes be transcribed into different isoforms [32]). As a consequence, we know little about the antibody reactivity of most *P. vivax* proteins and most studies so far have focused on a handful of candidate proteins selected for their biological role (*e.g.*, in red blood invasion) [27,33,34]. A few large screens have been conducted using protein arrays, but the difficulty and costs of synthesizing *Plasmodium* proteins have limited their scope to, at best, a few hundred genes that are expressed mainly in blood stage parasites [35–37]. High-density peptide arrays are an exciting alternative for simultaneously characterizing antibody responses to thousands of antigens and, potentially, for identifying their specific epitopes [38]. The synthesis of millions of short peptides (typically 10–20 amino acid long capturing linear B-cell epitopes [39,40]) is now possible, enabling screening of the entire protein repertoire of pathogens. Such peptide array technology has notably been successfully applied to *P. falciparum* to i) identify linear immunogenic B-cell epitopes in individuals naturally exposed to malaria [41–43], ii) measure serological responses post-vaccination [44,45], and iii) characterize the strain specificity of antibody responses [44,46].

Here, we describe the design and analysis of a proteome-wide *P. vivax* high-density peptide array and characterize the antibody response of Cambodian individuals during a *P. vivax* infection. We identified 283 proteins that elicit antibody responses across participants, including proteins known for their role in red blood cell invasion as well as many proteins with unknown functions that could play important roles in host/parasite interactions.

## Materials and methods

### Study population

Ten serum samples were collected in 2014 during a drug efficacy study in the Ratanakiri Province in Northeastern Cambodia [47]. Febrile patients with a positive non-falciparum rapid diagnostic test were enrolled and, after confirmation by PCR that the infection was caused solely by *P. vivax*, serum was collected from each participant before antimalarial treatment (**S1 Table**). In addition, we analyzed sera from five Cambodian individuals, enrolled in a longitudinal study in the Mondolkiri Province [48], that were infected with *P. vivax* but were asymptomatic at the time of the collection (**S1 Table**). As negative controls, we analyzed serum from ten North American individuals living in Baltimore (USA) and with no history of malaria exposure, no travel to or residence in a country where malaria is endemic, no history of sickle cell trait, no vaccines received in the past year, no current acute illness, and no history of fever in the three days prior to blood draw.

### Ethics statement

All Cambodian samples were obtained after written informed consent from the participants, and the analyses were approved by the National Ethic Committee of the Cambodian Ministry of Health (#038NECHR, 239NECHR and 057-NECHR). Serum collection from the North American individuals was approved by the University of Maryland, Baltimore Institutional Review Board (Protocol # HP-00071211).

### High-density peptide array design and data generation

We designed a high-density array containing 4.2 million peptides covering the entire amino acid sequence of all protein-coding genes annotated in the *P. vivax* P01 genome [49] (retrieved from PlasmoDB version 37). Briefly, we bioinformatically split the amino acid sequences from all annotated *P. vivax* protein-coding genes into 16 amino acid peptides overlapping the next peptide in the protein sequence by 15 amino acids (Fig 1A and **S1 Fig**). After removing redundant peptides (*i.e.*, we kept only one instance of a peptide found in multiple sequences), we analyzed a total of 4,168,042 non-redundant 16 amino acid long peptides.

**Fig 1:**
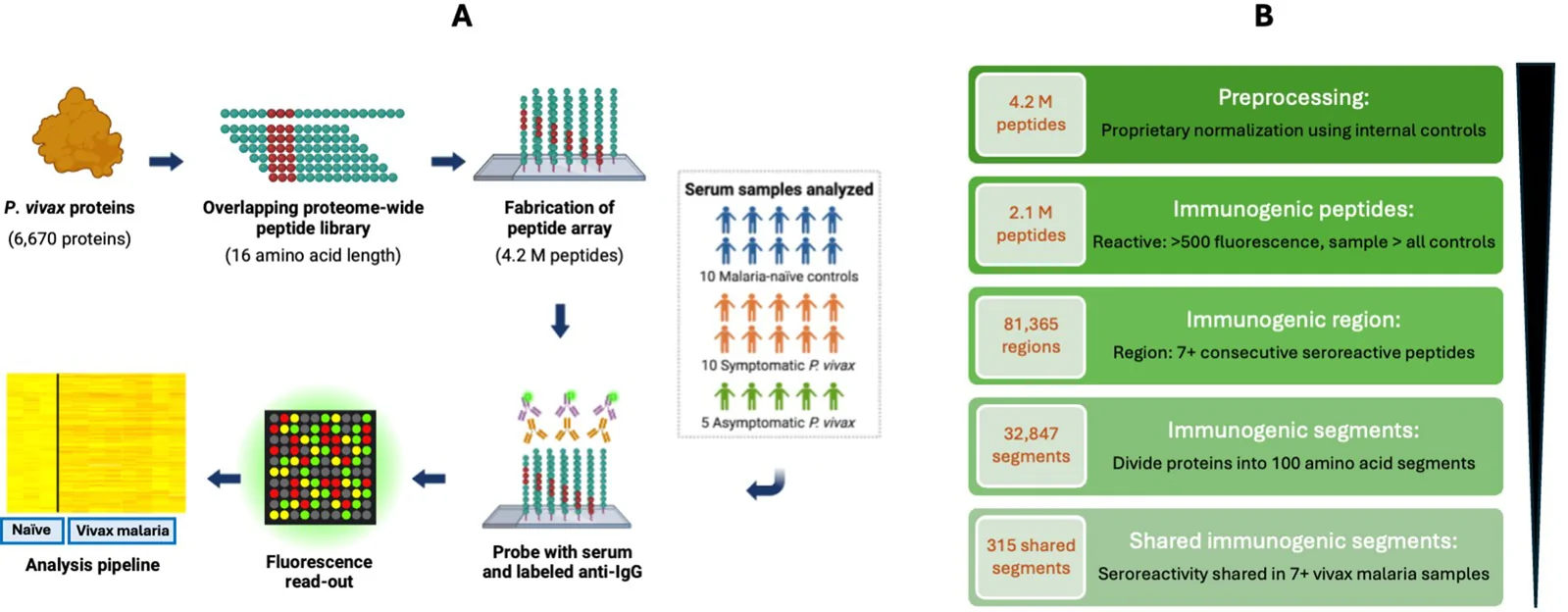
(A) Summary of the peptide array design and data generation. Each *P. vivax* protein was bioinformatically split into every possible 16 amino acid peptide, and all non-redundant peptides were printed on a high-density array. Serum from one individual was then added to the array and IgG bound to a specific peptide detected using a fluorescently-labeled secondary antibody. Figure created with BioRender. **(B)** Overview of the analytical pipeline used to determine IgG antibody binding. The numbers indicate the total number of features across all ten patients (i.e., not per individual).

Microarrays were synthesized with a Nimble Therapeutics Maskless Array Synthesizer (MAS) by light-directed solid-phase peptide synthesis using an amino-functionalized support (Greiner Bio-One) coupled with a 6-aminohexanoic acid linker and amino acid derivatives carrying a photosensitive 2- (2-nitrophenyl) propyloxycarbonyl (NPPOC) protection group (Orgentis Chemicals). Amino acids (final concentration 20 mM) were pre-mixed for 10 min in N,N-Dimethylformamide (DMF, Sigma Aldrich) with N,N,N’,N’-Tetramethyl-O- (1H-benzotriazol-1-yl)uronium-hexafluorophosphate (HBTU, Protein Technologies, Inc.; final concentration 20 mM) as an activator, 6-Chloro-1-hydroxybenzotriazole (6-Cl-HOBt, Protein Technologies, Inc.; final concentration 20 mM) to suppress racemization, and N,N-Diisopropylethylamine (DIPEA, Sigma Aldrich; final concentration 31mM) as base. Activated amino acids were then coupled to the array surface for 3 min. Following each coupling step, the microarray was washed with N-methyl-2-pyrrolidone (NMP, VWR International), and site-specific cleavage of the NPPOC protection group was accomplished by irradiation of an image created by a Digital Micro-Mirror Device (Texas Instruments), projecting 365 nm wavelength light. Coupling cycles were repeated to synthesize the full in silico-generated peptide library.

Prior to sample binding, final removal of side-chain protecting groups was performed in 95% trifluoroacetic acid (TFA, Sigma Aldrich), 0.5% Triisopropylsilane (TIPS, TCI Chemicals) for 30 min. Arrays were incubated twice in methanol for 30 s and rinsed four times with reagent-grade water (Ricca Chemical Co.). Arrays were washed for 1 min in TBST (1 × TBS, 0.05% Tween-20), washed 2× for 1 min in TBS, and exposed to a final wash for 30 s in reagent-grade water.

For each sample, 40 µl of serum were diluted 1:100 in binding buffer (0.01M Tris-Cl, pH 7.4, 1% alkali-soluble casein, 0.05% Tween-20) and bound to one array overnight at 4°C. After sample binding, the arrays were washed three times in wash buffer (1 × TBS, 0.05% Tween-20), 10 min per wash. Binding of human IgG was detected via Alexa Fluor 647-conjugated goat anti-human IgG secondary antibody (Jackson ImmunoResearch). The secondary antibody was diluted 1:10,000 (final concentration 0.1 ng/µl) in secondary binding buffer (1x TBS, 1% alkali-soluble casein, 0.05% Tween-20). Arrays were incubated with secondary antibody for 3 h at room temperature, then washed three times in wash buffer (10 min per wash), washed for 30 sec in reagent-grade water, and then dried by spinning in a microcentrifuge equipped with an array holder. Fluorescent signal of the secondary antibody was detected by scanning at 635 nm at 2 µm resolution using an Innopsys 1100AL microarray scanner (Innopsys Inc.). Scanned array images were analyzed with proprietary Nimble Therapeutics software to extract fluorescence intensity values for each peptide (Fig 1A).

### Determination of the serological response to *P. vivax* peptides

To identify *P. vivax* antigens among *P. vivax*-infected Cambodian adults, we used the analytical procedure summarized on Fig 1B. We first considered, separately for each infected individual serum sample, whether the fluorescence intensity (FI) determined for a given peptide was greater than the maximum FI observed across all North American controls. To avoid including very low signals, we (arbitrarily) only considered peptides with FI greater than 500. Since each 16 amino acid peptide shares 15 amino acids with the peptides immediately preceding and following it in a given protein-coding sequence, we considered, as potentially antigenic, regions of proteins with at least seven consecutive peptides with signals greater than those of the malaria-naïve controls. The choice of seven consecutive peptides (resulting in a shared examined region of 10 amino acids, **S1 Fig**) was driven by the average length of reported linear B-cell epitopes [39,40,50]) and not statistical considerations (statistical significance was rigorously determined later in the analyses, see below).

Finally, to allow for interindividual variability in the position of an antigenic region, we calculated the number of patients that were seropositive (*i.e.*, >=7 consecutive positive peptides) per 100 amino acid non-overlapping window across all proteins.

To rigorously assess statistical significance and identify antigenic proteins, we used a Poisson distribution to calculate the probability of observing by chance X patients seropositive for the same segment of 100 amino acid given the total number of potentially antigenic segments observed in one patient (**S2 Table**). Using this approach, we determined that the number of 100 amino acid segments identified as antigenic in seven or more of the ten patients was > 20 fold greater than the number expected by chance alone (*i.e.*, corresponding to an FDR < 0.05) and we considered these segments as antigenic for the rest of the analyses.

### Intrinsically disordered regions and antigenic segments

To determine whether intrinsically disordered regions (IDRs) were disproportionally identified as antigenic on the peptide array, we first used the Predictors Of Natural Disordered Regions (PONDR) [51] to identify IDRs in all annotated *P. vivax* proteins using custom scripts. We then split each protein into the same 100 amino acid segments as described above and considered a segment as disordered if >50% of its amino acids were predicted as disordered (*i.e.*, the VL-XT score was greater than 0.5). We then compared whether disordered segments were enriched within antigenic segments using a χ-square test.

### Repeated peptides and antigenic segments

16 amino acid peptides present in multiple proteins, or multiple times in the same protein, were only printed once on the array. To determine if repeated peptides were disproportionally identified as antigenic, we compared whether segments containing >25% or >50% repeated peptides were enriched within the antigenic segments using a χ-square test.

### Amino acid composition of antigenic segments

To test if specific peptide motifs were disproportionally represented among antigenic segments, we calculated the number of all possible 5 amino acid peptides occurring in antigenic segments vs non-antigenic segments and compared their frequencies using a Fisher’s exact test.

### Association between antigenicity and parasite mRNA expression in Cambodian individuals

To evaluate the association between the level of expression of a given protein and its antibody reactivity, we determined the average level of mRNA expression of all proteins in blood-stage parasites using bulk RNA-seq data generated from the same ten symptomatic *P. vivax* patients analyzed on the peptide array and collected at the same time [52]. Briefly, we reanalyzed the RNA-seq reads previously generated (BioProject: PRJNA378759) by mapping them with Hisat2 [53], first onto the human genome (NCBI Hg38 assembly) and then, for all the reads that did not align to the human genome, to the *P. vivax* genome (PlasmoDB-v68). After removing PCR duplicates using Picard, we calculated, for each patient, the number of reads mapped to each *P. vivax* gene. The average expression across all patients of each gene was then calculated after normalizing the counts obtained per patient to counts per million (CPM).

In order to determine if mRNA expression was statistically associated with antibody reactivity, we ranked all *P. vivax* genes tested on the peptide array based on their average mRNA expression and calculated, for 100 successive bins of ~60 genes, the number of proteins that were immunoreactive in seven or more samples. We also tested whether the expression of a gene was statistically correlated with its seroreactivity using logistic regression.

### Determination of complexity of infection and its association with antigenicity

We used whole genome sequence data generated from the parasites isolated from all symptomatic infections (and collected at the same time as the serum samples) [47] to determine the complexity of each infection. Briefly, we used Hisat2 with default parameters to map the reads to the P01 reference genome (version 67) and used GATK [54] to call nucleotide variants, excluding telomeric regions and multigene families. We then used the R package moimix [55] to estimate polyclonality, with infections with a Fws >= 0.95 being considered as monoclonal and those with Fws < 0.95 being considered as polyclonal. We then tested whether polyclonal infections displayed antibody binding to more *P. vivax* proteins than monoclonal infections using a Fisher exact test.

## Results and discussion

### Characterization of the seroreactivity of *P. vivax* proteins across symptomatic Cambodian individuals

We designed an ultra-dense peptide array containing 4.2 million peptides of 16 amino acids that cover the entire protein sequences of all *P. vivax* protein-coding genes (Fig 1 and **S1 Fig**, see Materials and Method for details). We first probed these arrays with i) sera from ten vivax malaria Cambodian patients and ii) sera from ten malaria-naïve individuals and used a fluorescent secondary antibody against IgG to measure the individuals’ antibody binding to each peptide. Out of the 4.2 million *P. vivax* peptides, 96,140 – 405,964 peptides (2–9%) per patient displayed a fluorescence intensity greater than the fluorescence measured in any of the ten malaria-naïve individuals and were further considered. Each 16 amino acid peptide on the array overlaps the next peptide in the protein sequence by 15 amino acids (**S1 Fig**). Given that a linear B-cell epitope is typically longer than 10 amino acids [40,50,56], we postulated that antibody binding to a specific protein should be detectable across multiple consecutive peptides. We therefore restricted our analyses to sections of proteins with, in a given patient, at least seven consecutive peptides each with a signal greater than the signals observed in all malaria-naïve individuals, corresponding to an epitope length of 10 amino acids (**S1 Fig**) (see Materials and Methods). We thus identified 3,332 – 16,197 putative immunoreactive regions per vivax malaria patient for a total of 81,362 putative immunoreactive regions across all patients (Fig 1B). The fluorescence intensities detected in these putative immunoreactive regions were, quantitatively, relatively similar (typically with a signal intensity of 5,000–20,000). This observation contrasts with the different affinities and/or concentrations expected for natural antibodies targeting different antigens and likely indicated that the signal measured with the peptide array was only semiquantitative, either because of saturation of the fluorescence signal or data normalization. For the remaining analyses, we therefore exclusively focused on qualitative analyses and comparisons of the signals measured in infected individuals and controls.

We observed large variations in the sizes of the putative immunogenic regions, with a mean length of 10 peptides (IQR = 8–11) (**S2 Fig**) but with a few regions containing more than 100 consecutive putative immunoreactive peptides. Interestingly, the largest putative immunogenic region (142–145 amino acids long) was detected in the circumsporozoite protein (CSP, PVP01_0835600), one of the most studied *Plasmodium* antigenic proteins [57], and corresponded to a highly repetitive domain (see also below). Other proteins with very large immunoreactive domains included the surface protein P113 [58] (PVP01_1328200, 71–91 AA) and the asparagine-rich merozoite protein ARMA [59,60] (PVP01_0937100, 99 AA). For many proteins, we observed several discontinuous putative antigenic regions (with an average of 5.7 regions identified in at least one patient per protein). One possible explanation for these multiple putative antigenic regions observed within one protein is that distant peptides (in the protein sequence) are in close proximity in the folded protein and represent a single 3D structure recognized by the host immune system (see also discussion of MSP1 below). Alternatively, this observation could reflect that different antibodies specifically bind to different domains of the same protein.

While putative immunoreactive regions often overlapped among patients, many regions were not identified as seroreactive in all patients, or their exact boundaries differed between patients (see, e.g., **S3 Fig**), suggesting that slightly different regions of the same protein might be immunogenic in different patients [61]. Therefore, to identify proteins that were immunogenic across multiple patients while accounting for interindividual variations, we considered signals by non-overlapping 100 amino acid segments (see Materials and Methods). Finally, we rigorously evaluated statistical significance by comparing the number of 100 amino acid segments that were deemed seroreactive in X patients with the number expected by chance under a Poisson distribution and determined that segments that were immunoreactive in seven or more individuals were unlikely to be observed by chance only (FDR < 0.05, **S2 Table**) and were considered immunogenic. Overall, we identified a total of 315 immunogenic segments from 283 protein-coding genes (**Table 1**, **S3 Table**). Each of the ten patients carried antibodies to 153–281 of these proteins (**S4 Fig**), with no clear associations between the number of immunogenic proteins identified in one patient and the complexity of infection (p = 0.74) or parasitemia (p = 0.68) at the time of collection. Out of the 283 immunogenic proteins identified here, 210 (74%) have not been examined in previous large seroreactivity screens [34,62], highlighting the benefits of a proteome-wide, agnostic, approach (**S3 Table**). Conversely, some of the proteins previously reported as immunogenic did not reach significance in our study (**S5 Fig**), although this discrepancy could be due to different cutoffs used (e.g., threshold of 10% positive patients in [62] vs. 7/10 here) rather than the probe design (linear peptides vs. recombinant proteins) or genuine population differences. Note in this regards that the analytical pipeline implemented in this study is designed to identify “shared” immunogenic signals across patients and might miss epitopes that are polymorphic among *P. vivax* parasites (*e.g.*, if the peptides printed differ from the sequences bound by an antibody due to amino acid polymorphisms).

**Table 1:** Antigenic *P. vivax* proteins detected in nine or ten of the symptomatic vivax malaria patients (the complete list of antigenic proteins in provided in S3 Table).

ID	Gene name	Description	No. patients reactive
PVP01_0418400	MSP5	merozoite surface protein 5	10
PVP01_0505600	GAMA	GPI-anchored micronemal antigen	10
PVP01_0712900	---	conserved Plasmodium protein, unknown function	10
PVP01_0000140	TRAG36	tryptophan-rich antigen, tryptophan-rich protein	9
PVP01_0106700	---	conserved Plasmodium protein, unknown function	9
PVP01_0422600	ETRAMP11.2	early transcribed membrane protein	9
PVP01_0508000	---	SPRY domain, putative	9
PVP01_0611100	---	conserved protein, unknown function	9
PVP01_0708400	---	helicase SKI2W, putative	9
PVP01_0722400	PABP2	polyadenylate-binding protein 2, putative	9
PVP01_0807800	PHD1	PHD finger protein PHD1, putative	9
PVP01_0902400	---	hypothetical protein	9
PVP01_0934200	AMA1	apical membrane antigen 1	9
PVP01_1103100	---	conserved protein, unknown function	9
PVP01_1109100	---	conserved Plasmodium protein, unknown function	9
PVP01_1201700	MAHRP2	membrane associated histidine-rich protein 2	9
PVP01_1264400	TREP	TRAP-like protein	9
PVP01_1403400	---	AAA domain-containing protein, putative	9
PVP01_1408600	---	TOM1-like protein, putative	9

### General features of the immunogenic proteins detected

Both linear and conformational B-cell epitopes have been shown to play roles in protective immunity and immune evasion and are suitable for diagnostic purposes. For example, linear epitopes within the unstructured repeats of CSP are critical for protection [63–65], while some linear and/or repetitive asparagine-rich repeats may contribute to immune evasion [66–69]. The high-density peptide array evaluates seroreactivity against linear epitopes and is limited in measuring the response to conformational epitopes (resulting from three-dimensional protein structures), unless a significant component of the conformational epitope is linear. To assess this limitation, we examined whether intrinsically disordered regions (IDRs) [70], which lack a predicted three-dimensional structure (but can sometimes change conformations based on interactions with other proteins or RNA molecules) were enriched among seroreactive segments. We observed that 37.3% of all immunoreactive segments were disordered, a 1.4-fold enrichment compared with the proportion of disordered regions in non-reactive segments (p = 2.2x10^-16^). Note however that 62.7% of the detected immunoreactive segments were derived from regions of the proteins with predicted tertiary structure, indicating that the immunogenic segments identified were not solely restricted to disordered domains.

Next, we evaluated whether repeated peptides (*i.e.*, peptides printed once on the array but present multiple times in one protein or in multiple proteins) were more likely to be seroreactive than were unique peptides. Among the 315 immunoreactive segments, only 10 (3.2%) contained >25% repeated peptides, very similar to the proportion among the non-seroreactive peptides (4.4%, p = 0.3), indicating that repeated peptides were not significantly more seroreactive in our dataset (similar results were obtained when considering segments with >50% repeated peptides).

We then examined whether specific amino acid motifs were over-represented in the immunoreactive segments. Three 5-mers, present in 17 different immunogenic proteins, were significantly enriched in immunoreactive segments: ANAAN, NAANA and AANAA (p = 3.59 x 10^-40^, p = 1.48 x 10^-26^, p = 3.39 x 10^-22^ respectively, Fisher’s exact test, **S6 Fig**). Interestingly, asparagine- and alanine-rich repeats have been reported in several *P. falciparum* antigens, possibly as “decoy epitopes” triggering a highly antigenic but non-protective immune response [69,71,72].

We also examined whether immunogenic *P. vivax* proteins were conserved among human *Plasmodium* parasites. Out of the 283 immunogenic proteins, 123 (44%) had no orthologous proteins in the *P. falciparum* genome (**S3 Table**). By comparison, only 36% of all *P. vivax* proteins assessed on the peptide array have no orthologous *P. falciparum* proteins (p = 0.0071). This enrichment suggests that *Plasmodium* proteins commonly exposed to the host immune system evolve faster and are more divergent, or that species-specific pathways are disproportionally exposed (see also the discussion of invasion genes below). Importantly, 78 of the 283 immunogenic *P. vivax* proteins identified have no orthologs in any of the malaria species typically infecting humans (*P. falciparum, P. knowlesi, P. malariae* and *P. ovale*) and these putative *P. vivax* antigens might therefore support the development of novel species-specific detection assays (e.g., RDTs) (**S3 Table**).

Finally, we examined whether the immunogenicity of a protein was associated with its level of expression. Since we lack comprehensive quantitative data on protein abundance, we used the mRNA expression levels determined by whole-blood RNA-seq, generated from the same symptomatic *P. vivax* infections at the same time, as a proxy [52]. While the level of mRNA expression determined from patient blood is influenced by the stage composition of each infection, we noted that proteins that were, on average, highly expressed by blood-stage parasites were more likely to be seroreactive (**S7 Fig**, p = 1.28 x 10^-15^), suggesting that the amount of a protein present during a blood stage infection may be one of the factors influencing the ability of the immune system to mount a humoral immune response (in addition to the protein localization, its time of expression or shielding).

### Many erythrocyte invasion proteins are highly immunogenic

Among the 283 immunogenic *P. vivax* proteins, many proteins known to be involved in erythrocyte invasion were detected (Fig 2). For example, Merozoite Surface Protein 5 (MSP5), Tryptophane-Rich Antigen 36 (TRAg36) and Apical Membrane Antigen 1 (AMA1) were among the most common antigenic proteins (**Table 1**).

**Fig 2:**
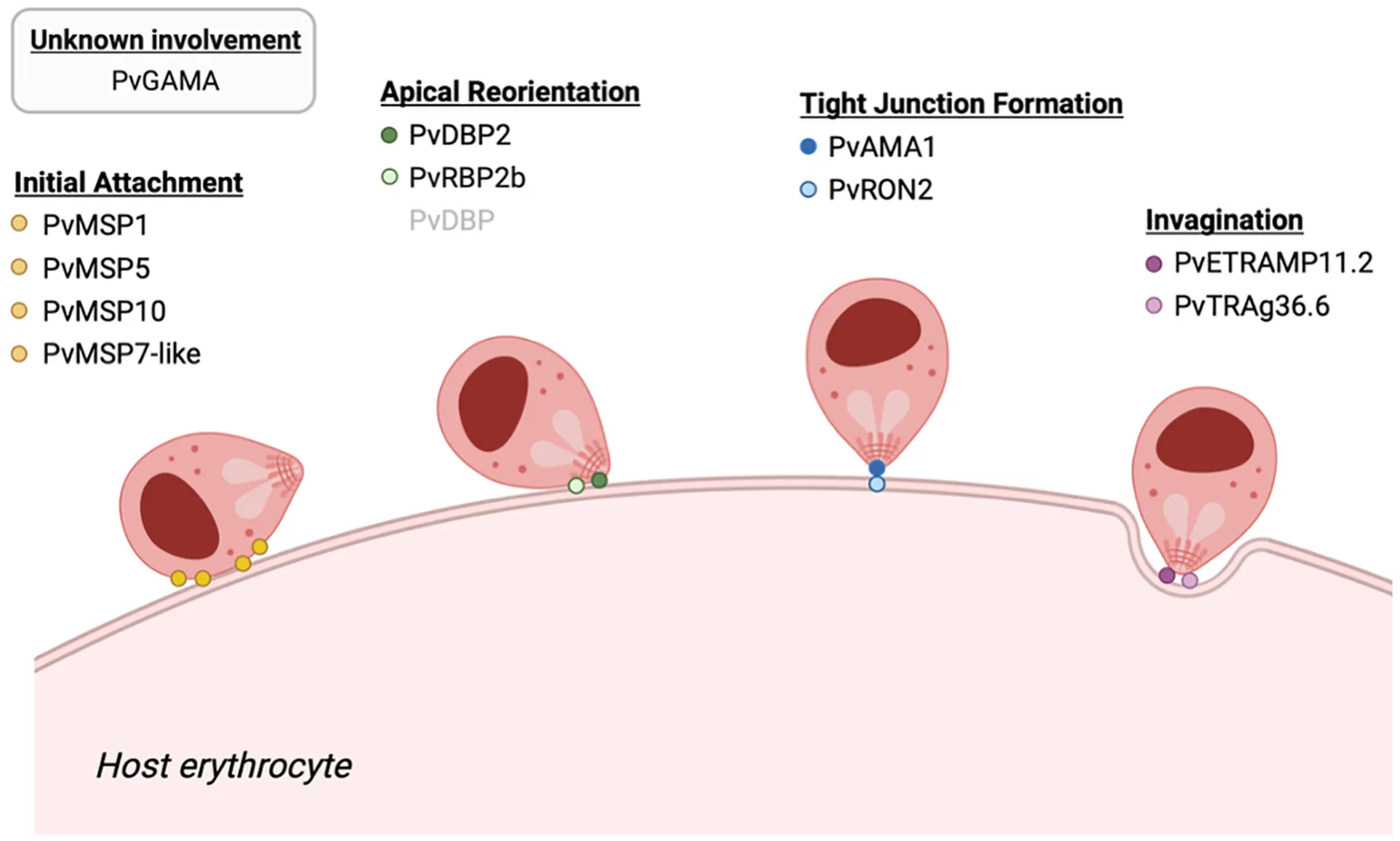
*P. vivax* proteins involved in erythrocyte invasion are highly immunogenic. The figure shows the proteins known to be involved at different stages of red blood cell invasion. Proteins deemed immunogenic on our high-density peptide array are indicated in black, while proteins that did not reach the significance threshold are indicated in grey. Figure created with BioRender.

Antibodies against MSP5 (PVP01_0418400) were detected in all ten patients, whereas antibodies against MSP10 (PVP01_1129100), MSP1 (PVP01_0728900), and MSP7-like protein (PVP01_1220300) were detected in more than seven patients (**S3 Table**, **S8 Fig**). Merozoite surface proteins are low affinity “adhesins” involved in the initial attachment of the parasite to erythrocytes prior to invasion [73,74]. Most of these proteins remain incompletely characterized, especially in *P. vivax,* but might act as part of complexes with other MSPs and additional proteins and are already present on the surface of the merozoites upon egress from the schizonts. Only MSP1 has been the focus of detailed serological studies in *P. vivax,* and the C-terminal domain (MSP1–42 kDa) has notably been shown to be highly antigenic [75–77], with antibodies against this region being detectable several months after one infection [78]. The signal detected on the peptide array overlapped with the 42 kDa region but appeared to be restricted to the highly polymorphic MSP1–33 kDa fragment rather than located in the MSP1–19 kDa region, the putative RBC binding domain and a major target of protective immunity [79] (Fig 3A). Interestingly, the two discontinuous 100-amino acid regions identified as antigenic on the peptide array are in close proximity in the predicted 3D structure of MSP1 and, possibly, capture a single, conformational, epitope (Fig 3A).

**Fig 3:**
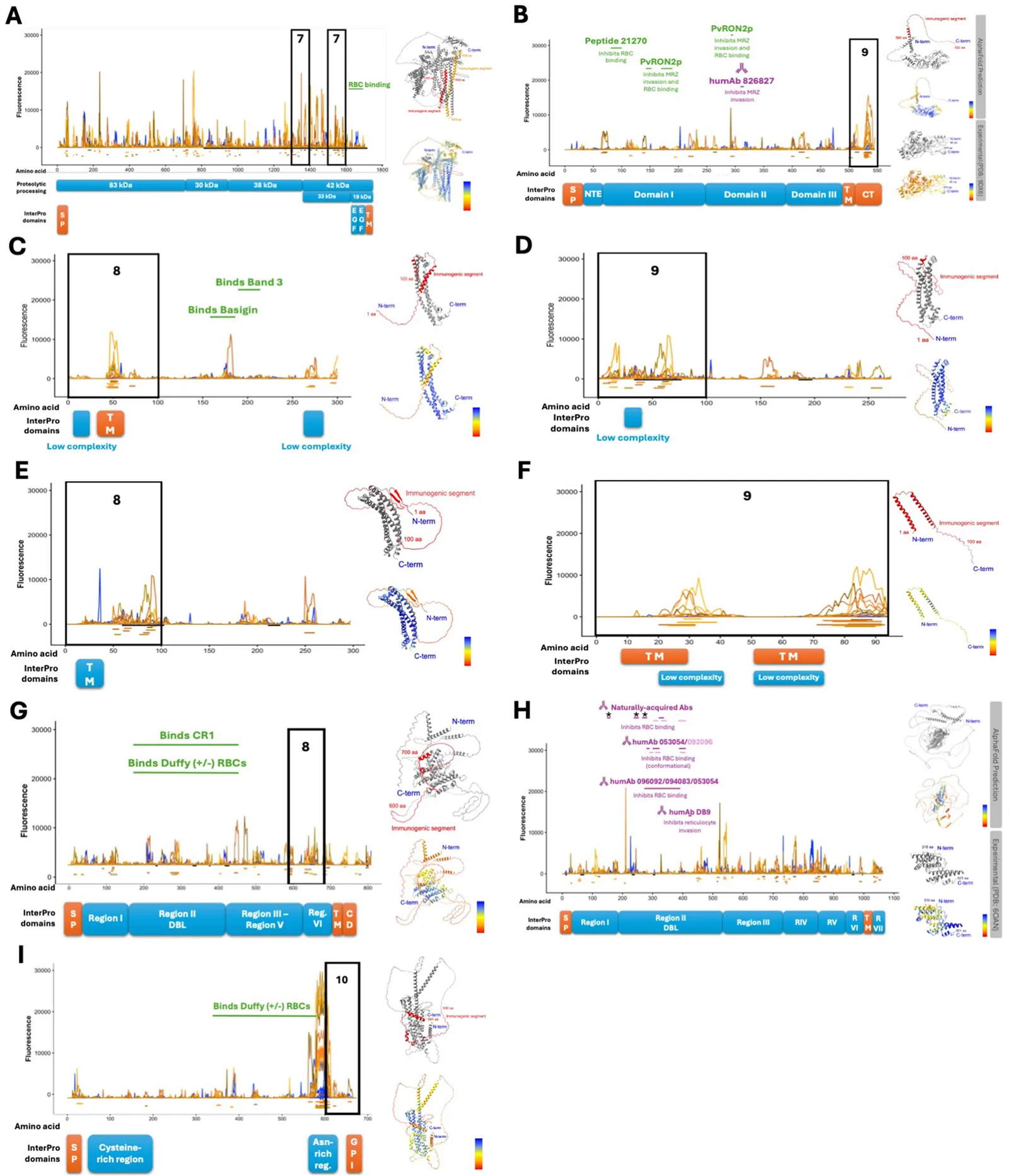
IgG antibody binding across *P. vivax* invasion proteins: (A) Merozoite surface protein 1 (MSP1, PVP01_0728900), (B) Apical membrane antigen 1 (AMA1, PVP01_0934200), (C) TRAg38 (PVP01_0503600), (D) TRAg36 (PVP01_0000140), (E) TRAg36.6/32 (PVP01_0000170), (F) ETRAMP11.2 (PVP01_0422600), (G) Duffy binding protein 2 (DBP2, PVP01_0102300), (H) Duffy binding protein (DBP, PVP01_0623800) and (I) GPI-anchored micronemal protein (GAMA, PVP01_0505600). Each panel shows the fluorescence detected (y-axis) at peptides organized according to the protein amino acid sequence (x-axis). The blue lines show the data for each malaria-naïve control (n = 10) and the yellow to red lines represent data for the symptomatic individuals (n = 10). The black horizontal bar below the sample lines indicates peptides that are found multiple times on the array (repeated peptides probed only once). Colored horizontal bars indicate consecutively antigenic peptide regions, colored by symptomatic individual. Each black box indicates a 100 amino acid segment that was found to be significantly antigenic, with the number of reactive symptomatic individuals denoted at the top (out of ten). The blue and orange boxes under each plot indicate functional regions and/or proteolytic domains. The green bars indicate regions of the protein known to be associated with RBC binding, while purple bars indicate binding locations of human antibodies with functional anti-parasite activity, colored by specific epitope. “*” denotes the location of DBP residues which resulted in loss of function in a rosetting assay after mutagenesis. The plot on the upper right displays the predicted 3D structures (based on AlphaFold), with the N- and C-terminus indicated and the antigenic segments represented in red and orange. The plot on the lower right displays the associated confidence intervals acquired from the AlphaFold prediction, with blue indicating high confidence and red indicating low confidence.

Peptides from the apical membrane antigen 1 (AMA1, PVP01_0934200) were recognized in nine of the ten patients and displayed antibody binding signal in the C-terminal region that overlapped with the cytoplasmic tail (CT) domain [80,81] (Fig 3B). AMA1 is a micronemal protein found on the apical end of merozoites that mediates the initiation of tight junction formation [82,83] (Fig 2). While it is only secreted from the microneme at the time of invasion [84], AMA1 is one of the best characterized *Plasmodium* antigens and is a leading candidate for monoclonal antibody therapy [85–87] and vaccine design [88]. Similar to MSP1, the location of antigenic signal detected by the peptide array was located in the C-terminal domain. In both *P. falciparum* and *P. vivax*, the 22 kDa cytoplasmic tail is carried into the host cell after cleavage of the AMA1 ectodomain, which is thought to facilitate evasion of protective anti-AMA1 immune responses, primarily targeting domain II [89] (Fig 3B). The binding partner of AMA1, the rhoptry neck protein 2 (RON2, PVP01_1255000) was also identified as immunogenic, with an antibody binding signal located at the N-terminus of the protein (**S9 Fig**). RON2 is a parasite protein that is stored in the rhoptry neck and injected into erythrocytes, before becoming embedded in the host membrane, where it serves as a ligand for AMA1 to initiate the tight junction formation and trigger erythrocyte invasion [84,90].

Several tryptophan-rich antigen (TRAg) genes were also among the most commonly seroreactive proteins, including TRAg36 (PVP01_0000140) and TRAg38 (PVP01_0503600) (Fig 3C-D), which may bind to Band 3 and basigin on the host erythrocyte [91–96], as well as TRAg32/36.6 (PVP01_0000170) and its binding partner ETRAMP11.2 (PVP01_0422600) [97] (Fig 3E-F). TRAgs are members of a multigene family present in all *Plasmodium* species but are significantly expanded in *Plasmodium vivax* [98]. While the exact role of these proteins remains unclear, several of these proteins are able to bind to RBCs, possibly via lipid binding [99] or directly to host proteins (possibly Band 3 and basigin [92,93]), and they have been shown to stimulate strong cellular and humoral immune responses in *P. vivax* exposed patients [98,100–102].Finally, we identified the Duffy binding protein 2 (DBP2, PVP01_0102300) [103], also referred to as erythrocyte binding protein (EBP or EBP2), among the most immunogenic proteins (**S3 Table**, Fig 3G). DBP2 may mediate a Duffy-independent invasion pathway [104], possibly through binding to complement receptor 1 [105]. The Duffy-binding protein (DBP, PVP01_0623800), one of the most studied *P. vivax* proteins and a critical reticulocyte invasion protein, did not reach significance in our analyses (Fig 3H). This lack of signal contrasts with previous studies that have clearly demonstrated humoral immunity against DBP [106–108] but may be explained by the main limitation of peptide arrays, which primarily identify linear epitopes: several studies have shown that antibodies to DBP bind mainly conformational epitopes [109–111] and are therefore unlikely to be captured by short amino acid peptides. Similarly, we failed to detect antibody binding signals for most Reticulocyte Binding Proteins (RBPs [112]), with the exception of RBP2b (PVP01_0800700, **S10 Fig**), which contrasts with the results of previous serological studies [113] but may be explained by the conformational epitopes of these proteins [112].

We also detected antibody-binding signal for one putative invasion protein that has received less attention in *P. vivax*: the GPI-anchored micronemal protein (GAMA, PVP01_0505600). Peptides of the protein were recognized by antibodies from all ten patients, with a clearly defined peak of reactivity centered on the asparagine-rich region at the C-terminus of the protein (**Table 1**, Fig 3I). While this protein has not been extensively studied in *P. vivax,* it is believed to play a role in reticulocyte invasion [114], possibly via interactions with MSP10 [115] and binding to Band 3 [116].

Overall, the identification of many key invasion genes among seroreactive *P. vivax* proteins is consistent with the localization of these proteins on the surface of the merozoites and, therefore, their exposure to the host immune system (while most *P. vivax* proteins are hidden inside the parasites and/or the RBCs and are less likely to be recognized). While protective antibodies have been reported for several of these invasion proteins, it is noteworthy that the signal of antibody binding detected among the Cambodian patients typically did not overlap with these protective antibody binding regions. One possible explanation for this observation is that the individuals analyzed here were experiencing a symptomatic episode of vivax malaria and, therefore, had not developed protective immunity against the parasites (but also see below) and that the signal identified reflected the humoral detection of these immunogenic proteins but, possibly, from domains implicated in immune evasion.

### SEC13 and other nucleoporins are enriched among immunogenic proteins

A second group of proteins, nucleoporins, which form pore complexes on the nuclear envelope of *Plasmodium* parasites [117], were significantly enriched among the proteins eliciting a humoral immune response (p = 1.37 x 10^-6^). We identified six nucleoporins that were commonly immunogenic among Cambodian patients: NUP313, NUP205, NUP138, NUP434, SEC13, and SEC31 (**S3 Table**, **S11 Fig**). This finding was somewhat surprising, as in contrast to invasion proteins that are displayed at the surface of the merozoites and therefore exposed to the host immune system, nucleoporins are believed to be hidden within the parasite, behind the RBC- and the parasitophorous vacuolar membranes. However, these proteins may also participate in other functions, with different cellular localizations that may expose them to the host immune system. Notably, SEC13 seems to be localized, at least in *P. falciparum* gametocytes and sporozoites, outside the nucleus and may be associated with proteins involved in secretion or invasion mechanisms [118]. Our data would be consistent with such a dual role and support further investigations of the function of these proteins.

### Some *P. vivax* hypothetical proteins are highly immunogenic

Half of the *P. vivax* genes encode proteins without functional annotation. Among the 283 highly immunogenic proteins, 104 fell within this category and were labeled solely as “conserved”, “hypothetical proteins” or “exported proteins” without further annotations (**S3 Table**). The identification of antibody-binding signals for these 104 proteins could indicate that they are located on the surface of merozoites, exported to the surface of the RBCs, or possibly secreted. One interesting example is PVP01_1201600, which is only annotated as an exported protein. This protein is only 298 amino acids long but displayed an antibody binding signal localized between the 75^th^ and 100^th^ amino acids and identified in eight out of the ten patients (**S3 Table**, Fig 4A). This gene has no ortholog in *P. falciparum* but is located, in *P. vivax*, immediately upstream of the membrane associated histidine-rich protein 2 (MAHRP2, PVP01_1201700) and is the 28^th^ most highly expressed gene by blood-stage parasites in our RNA-seq data (**S7 Fig**). This immunogenicity, combined with this high expression pattern, is fascinating and points to a unique role for this short protein during blood-stage infections and warrants further investigations. Similarly, we observed similar antibody-binding signals in PVP01_1103100, PVP01_1426900, PVP01_1271400, and PVP01_1030800 (Fig 4B-E), suggesting that these proteins may be particularly exposed to the host immune system and that their examination should be prioritized in functional studies.

**Fig 4:**
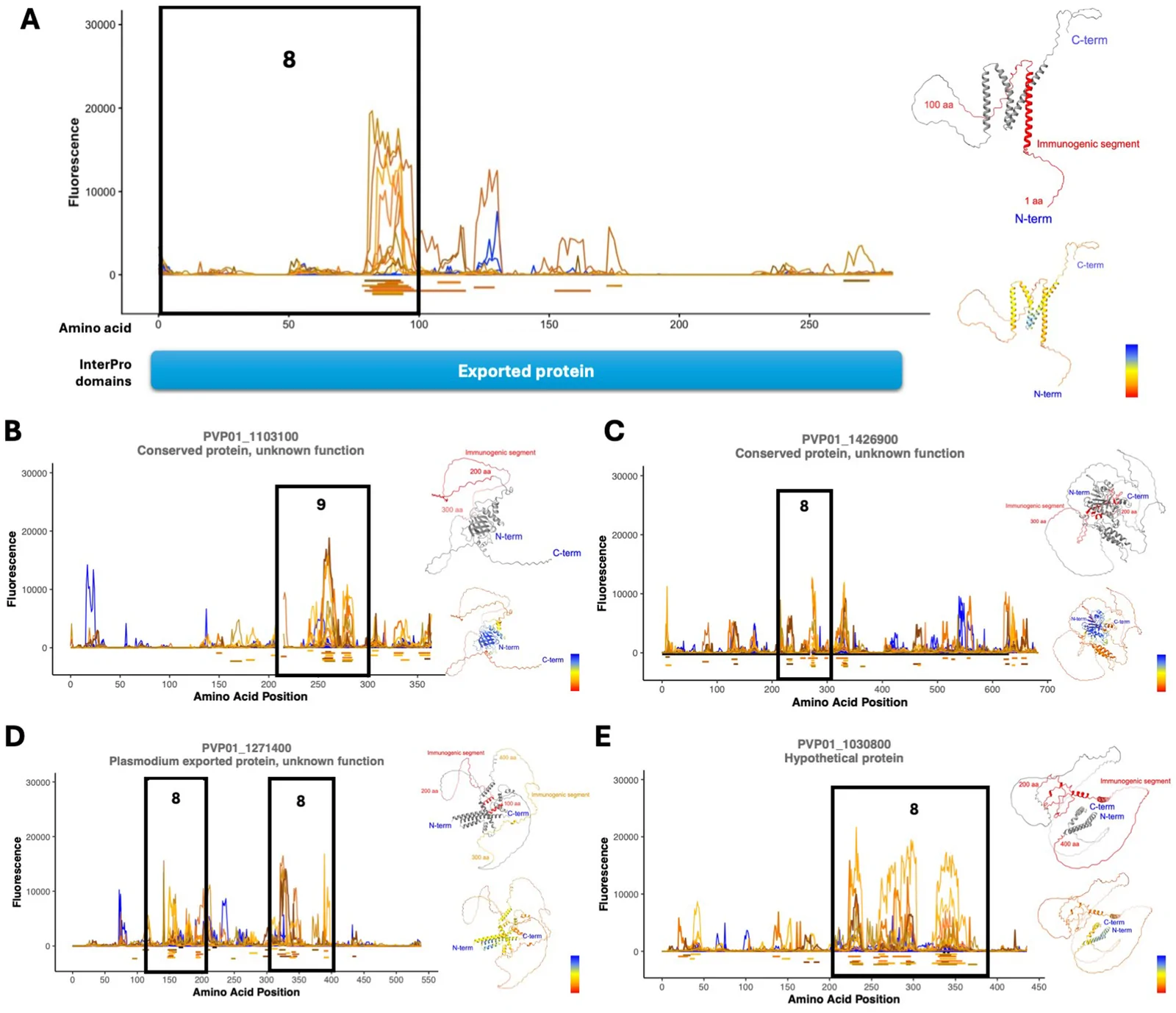
IgG antibody binding across hypothetical and unannotated *P. vivax* proteins. **(A)** PVP01_1201600, **(B)** PVP01_1103100, **(C)** PVP01_1426900, **(D)** PVP01_1271400 and **(E)** PVP01_1030800. See legend of Fig 3 for details.

### Low frequency of antibody binding to sporozoite peptides

The circumsporozoite protein (CSP, PVP01_0835600), which covers the entire surface of sporozoites [57] and is the target of the two current malaria vaccines in *P. falciparum* (RTS,S and R21 [119]), exhibited the longest stretch of seroreactive amino acids. However, CSP was recognized by antibodies in only five out of ten patients and therefore did not reach our significance threshold (**S3 Table**, **S12A Fig**). One possible explanation for this low frequency of anti-CSP antibodies is that many infections caused by *P. vivax* derive from the reactivation of hypnozoites and not directly from an infectious bite [120] and that some of the patients studied might not have been recently exposed to sporozoites.

To evaluate this possibility, we examined antibody binding to other key sporozoite proteins. TREP (PVP01_1264400) was the only sporozoite protein that reached the significance threshold, and for which antibodies were detected in nine of the ten patients (**S12B Fig**). In rodent parasites, the TREP protein is involved in the gliding motility of sporozoites and might therefore be exposed to, and recognized by, the host immune system [121]. In contrast, antibodies against TRAP (PVP01_1218700), another pre-erythrocytic vaccine candidate evaluated alongside CSP [122,123], and MAEBL (PVP01_0948400), a sporozoite adhesin essential notably for attachment to the mosquito salivary glands [124,125], were detected in only six and five patients, respectively (**S12C-D Fig**). Overall, antibody binding to sporozoite proteins reached significance less frequently than binding to peptides derived from proteins involved in erythrocyte invasion, consistent with the low immunogenicity of pre-erythrocytic stages and possibly reflecting the major differences in the number of parasites from these stages. Antibodies from two of the ten patients did not recognize either CSP, TRAP or MAEBL (but were positive for TREP). One could speculate that these patients’ infections were relapses caused by the reactivation of dormant liver-stage parasites and that these individuals may not have been exposed to an infectious bite for several months, explaining their absence of detectable antibody reactivity to sporozoite proteins. Additional analyses, including a larger cohort of patients and longitudinal data to evaluate antibody decay over time, will be necessary to determine whether antibody reactivity to these proteins could be informative for discriminating relapses from new infections. It would also be interesting to robustly characterize the expression (or lack thereof) of TREP in sporozoites and blood-stage *P. vivax* parasites, as, if TREP is indeed exclusively expressed in sporozoites, measurement of seroreactivity against TREP may allow for more robust detection of infective bites and assessment of ongoing transmission.

### The antibody binding profiles of asymptomatic vivax malaria individuals differ from those of patients

In addition to symptomatic patients, we also probed the peptide array with sera from five asymptomatic Cambodian individuals infected with *P. vivax.* The smaller sample size prevented us from rigorously assessing the statistical significance and determining with confidence which proteins elicited a humoral immune response in these individuals (the false discovery rate corresponding to detection of a region in all five asymptomatic individuals was only 0.6). Nonetheless, we observed 483 *P. vivax* segments (from 414 proteins) whose fluorescence signal was greater than that of all malaria-naïve individuals in all five asymptomatic individuals tested (**S4 Table**). Interestingly, this number is 5-fold greater than the average number of regions significantly immunoreactive in five random symptomatic individuals: when we randomly sampled five patients out of the ten symptomatic individuals screened on the peptide array, we identified only 29–146 regions (with a mean of 90) that were significant in all of them (compared with the 483 segments observed in five asymptomatic individuals). While this finding needs to be validated in a much larger cohort, it could indicate that asymptomatic individuals have antibodies against a broader repertoire of *P. vivax* proteins than symptomatic individuals. One possible explanation for this observation is that this broader repertoire of antibodies against *P. vivax* proteins may help individuals control the infection and prevent the development of malaria symptoms, although it is important to reiterate here that the peptide array does not provide any information on the protective nature of the antibodies detected. Indeed, examination of the profile of antibody binding along the MSP1, AMA1 or DBP protein sequences failed to reveal a significant signal overlapping the binding location of known protective antibodies (**S13 Fig**). An alternative, but not exclusive, explanation for the larger number of recognized proteins in asymptomatic individuals is that it may result from longer exposure to malaria parasites and the development of a broader, but not necessarily protective, antibody response: while we do not have any information regarding the time of infection of any of the individuals, it is likely that asymptomatic individuals have chronic and long-sustained infections, whereas the symptomatic individuals may result from a recent, uncontrolled infection. Further studies, including longitudinal monitoring of the antibody responses of both asymptomatic and symptomatic individuals, will be necessary to differentiate between these hypotheses.

The small sample size of the asymptomatic cohort analyzed here prevented us from rigorously determining which specific proteins were statistically seroreactive in asymptomatic individuals. However, among the 483 protein segments that elicited antibody responses in all five asymptomatic individuals, we observed 69 PIR segments (**S4 Table**). PIR genes are members of a highly variable multigene family that are possibly responsible for antigenic variation in *P. vivax* [126]. Out of the 69 PIR segments that were immunogenic in all asymptomatic individuals, only seven were deemed antigenic in symptomatic patients (*i.e.*, detected in seven or more patients). In fact, we detected antibodies against twice as many PIR proteins, proportionally, in asymptomatic individuals than in symptomatic patients (16.7% vs 7.1%, p = 0.0006). While this result needs to be confirmed, the increased frequency of antibodies elicited against PIR proteins observed in asymptomatic patients could suggest that these antibodies play a protective role against clinical malaria.

## Conclusion and limitations of the study

We identified 283 proteins that were immunogenic among symptomatic vivax patients and that included most of the proteins known to be involved in RBC invasion. While the domains identified as immunogenic in our study differed from domains previously reported to be associated with protective immunity in DBP, AMA1 or MSP1, immunogenic proteins identified here could provide new targets for vaccine development (although note that some weakly immunogenic proteins, such as DBP, can generate potent vaccine-elicited antibodies [102]). In particular, we observed immunoreactivity of putative invasion proteins, several nucleoporins and uncharacterized proteins that should be further investigated for their roles during blood-stage infections. Our analyses also revealed a unique binding pattern of antibodies targeting PIR proteins in asymptomatic individuals, which may be associated with protection against clinical vivax malaria. Several limitations of this study will need to be addressed in future studies. First, only a small number of infected individuals were analyzed here, and it will be important to assess whether our findings hold true in other endemic settings (with different transmission intensity, different patterns of *P. vivax* genetic diversity and with individuals with different HLA backgrounds). Second, the analyses described rely on the characterization of antibody binding from serum of vivax malaria patients who likely have been infected for different durations and have variable previous exposure. It would therefore be important to characterize the dynamics of the antibody response for promising antigenic proteins in longitudinal cohorts. Finally, field and laboratory studies will be required to evaluate if the proteins identified here induce protective antibodies and might indeed be used for vaccine development. Overall, the analyses presented here provide a unique, agnostic, and proteome-wide, perspective on antigenic *P. vivax* proteins and will constitute an important resource for the malaria community to develop new tools for the detection and elimination of this important human pathogen.

## Supporting information

S1 Fig Schematic showing consecutive peptides of 16 amino acid (green horizontal bars) overlapping by 15 amino acids. Analysis of seven consecutive peptides leads to a shared region of 10 amino acids (purple box).
(TIF)

S2 Fig Distribution of the number of consecutive seroreactive peptides detected on the peptide array. Note that the x axis is shortened for display purposes.
(TIF)

S3 Fig Example of one protein (MSP5, PVP01_0418400) with extensive inter-individual variations in antibody binding among patients. The x-axis represents the position of consecutive peptides assessed by the high-density array and the y-axis the fluorescence measured at each peptide. The yellow, orange and brown lines show the IgG antibody binding for three Cambodian patients, while the blue lines represent data from all malaria-naïve individuals (n = 10). The horizontal lines under the plot show locations where seven or more consecutive peptides have a signal greater than all malaria-naïve controls (colored by patient). The blue and orange boxes under each plot indicate annotated functional regions.
(TIF)

S4 Fig Number of immunogenic proteins recognized per patient. The left plot shows the number of proteins recognized in each patient (out of the 283 significantly immunogenic proteins), colored according to the complexity of each infection (light blue – monoclonal, dark blue – polyclonal). The right figure shows an upset plot indicating the number of proteins recognized by different combinations of patients.
(TIF)

S5 Fig Venn diagram showing the number of seroreactive *P. vivax* proteins identified in this study compared to Longley et al., 2017 and Chuquiyauri et al., 2015. Note that the enrollment criteria, number of proteins tested, definition of seroreactivity and methodology differ between these studies.
(TIF)

S6 Fig Distribution of all 5 amino acid motifs across antigenic segments (y-axis) and non-antigenic segments (x-axis). (TIF)

S7 Fig Association between the average level of mRNA expression of a gene in blood stage parasites and protein antigenicity. The x-axis shows 100 bins of 66 *P. vivax* genes grouped according to their average mRNA expression in blood stage parasites (black line, right y-axis) and ranked from left (most expressed genes) to right (least expressed genes). The vertical bars indicate the number of antigenic proteins in each bin (left y-axis).
(TIF)

S8 Fig Antigenic profiles of merozoite surface proteins (MSPs). See legend of Fig 3 for details.
(TIF)

S9 Fig Antigenic profile across the rhoptry neck protein 2 (RON2, PVP01_1255000). See legend of Fig 3 for details.
(TIF)

S10 Fig Antigenic profiles of reticulocyte binding proteins (RBPs) See legend of Fig 3 for details. (TIF)

S11 Fig Antigenic profiles of nucleoporins. See legend of Fig 3 for details.
(TIF)

S12 Fig Antigenic profiles across selected sporozoite-related proteins. See legend of Fig 3 for details.
(TIF)

S13 Fig Antigenic profiles of the asymptomatic individuals across selected invasion proteins with known protective antibodies. See legend of Fig 3 for details.
(TIF)

S1 Table Characteristics of the symptomatic (n = 10) and asymptomatic (n = 5) individuals analyzed on the peptide array. (TIF)

S2 Table Determination of the false discovery rate (FDR). The table indicates the number of observed and expected (under a Poisson distribution) immunogenic segments shared across X symptomatic individuals and the corresponding FDR.
(TIF)

S3 Table Immunogenicity of all *P. vivax* proteins. The table indicates, for each protein-coding gene, the number of patients with antibody binding in our analyses (out of ten). Proteins recognized in seven or more patients were deemed immunogenic and are indicated in bold. The table also includes, for comparison, the results from Chuquiyauri et al., Am. J. Trop. Med. Hyg. (2015) and Longley et al., PLoS Negl. Trop. Dis. (2017) (NA: not tested, Yes: immunogenic, No: not immunogenic), as well as the orthologs in other *Plasmodium* species.
(TIF)

S4 Table *P. vivax* proteins putatively reactive in all asymptomatic individuals (n = 5). (TIF)
